# A Compact Soft Robotic Wrist Brace With Origami Actuators

**DOI:** 10.3389/frobt.2021.614623

**Published:** 2021-03-25

**Authors:** Sicong Liu, Zhonggui Fang, Jianhui Liu, Kailuan Tang, Jianwen Luo, Juan Yi, Xinyao Hu, Zheng Wang

**Affiliations:** ^1^ Shenzhen Key Laboratory of Biomimetic Robotics and Intelligent Systems, Department of Mechanical and Energy Engineering, Southern University of Science and Technology, Shenzhen, China; ^2^ Guangdong Provincial Key Laboratory of Human-Augmentation and Rehabilitation Robotics in Universities, Southern University of Science and Technology, Shenzhen, China; ^3^ Department of Mechanical and Energy Engineering, Southern University of Science and Technology, Shenzhen, China; ^4^ Institute of Human Factors and Ergonomics, College of Mechatronics and Control Engineering, Shenzhen University, Shenzhen, China

**Keywords:** origami, soft actuator, wearable, soft robot, recovery device

## Abstract

Wrist disability caused by a series of diseases or injuries hinders the patient’s capability to perform activities of daily living (ADL). Rehabilitation devices for the wrist motor function have gained popularity among clinics and researchers due to the convenience of self-rehabilitation. The inherent compliance of soft robots enabled safe human-robot interaction and light-weight characteristics, providing new possibilities to develop wearable devices. Compared with the conventional apparatus, soft robotic wearable rehabilitation devices showed advantages in flexibility, cost, and comfort. In this work, a compact and low-profile soft robotic wrist brace was proposed by directly integrating eight soft origami-patterned actuators on the commercially available wrist brace. The linear motion of the actuators was defined by their origami pattern. The extensions of the actuators were constrained by the brace fabrics, deriving the motions of the wrist joint, i.e., extension/flexion, ulnar/radial deviation. The soft actuators were made of ethylene-vinyl acetate by blow molding, achieving mass-production capability, low cost, and high repeatability. The design and fabrication of the soft robotic wrist brace are presented in this work. The experiments on the range of motion, output force, wearing position adaptivity, and performance under disturbance have been carried out with results analyzed. The modular soft actuator approach of design and fabrication of the soft robotic wrist brace has a wide application potential in wearable devices.

## Introduction

Disease such as stroke causes upper limb motor impairment. The capability of the arm to perform activities of daily living (ADL) is limited due to the loss of motor control (Krakauer, 2005). The timely rehabilitation helps to induce neural plasticity and recovery, hence the motor recovery (Hendricks et al., 2002), by repetitive movement training or massed practice on the joints (Brewer et al., 2007; Martinez et al., 2013). Plasticity refers to the brain’s ability to reorganize itself, which can be stimulated through physical therapy (Celestino, 2003). A number of robotic rehabilitation devices have been developed and employed in the clinical research and therapy (Brewer et al., 2007). In comparison with the physiotherapist-assisted rehabilitation therapy, the robotic rehabilitation therapies showed promising advantages by providing automatic, haptic, high repetitions, longer duration, portable and adjustable customized therapies (Brewer et al., 2007; Ferreira et al., 2018). The benefits of the robot assisted rehabilitation therapies have led to increased interest in the development of rehabilitation robots. The human upper limb consists of complex skeletal structure, which includes shoulder complex, elbow complex, wrist joint, and fingers (Gull et al., 2020). The state-of-the-art robotic rehabilitation devices for upper limbs can be grouped into the desk-like systems, such as the commercially available Bi-Manu-Track (Brewer et al., 2007), and the wearable systems, such as the upper limb exoskeletons (Gull et al., 2020). The existing rehabilitation robots provide convenient therapy in a daily living environment but are still facing challenges in kinematic compatibility, discomfort, misalignment, and affordability on the way to broad application.

In recent years, soft robotics gained popularity among researchers due to merits in compliance and safe human-robot interaction. In particular, in the development of wearable rehabilitation robots, soft robotics have provided new solutions (Peng and Huang, 2019; Thalman and Artemiadis, 2020). Unlike the conventional devices that use electric motors and rigid linkages to constrain and guide the motions of the human joints, the soft wearables utilize soft actuators associated with flexible materials to repetitively move the joints, thus obtaining the effectiveness of rehabilitation. The inherent compliance of the soft robots enhances the wearing comfort, while achieving low profile, light weight, and low cost (Peng and Huang, 2019). The advantages of soft robotic wearable devices have paved the road for a broad application toward at-home rehabilitation (Polygerinos et al., 2015).

A series of soft robotic wearable rehabilitation (SRWR) devices have been proposed for hand (Polygerinos et al., 2015; Yap et al., 2015), wrist (Sasaki et al., 2005; Realmuto and Sanger, 2019), elbow (Xiloyannis et al., 2017), shoulder (O’Neill et al., 2017), knee (Baiden and Ivlev, 2013), and ankle (Park et al., 2014; Kwon et al., 2019). For integrated rehabilitation therapies, multijoint soft exoskeletons have been developed for upper (Nycz et al., 2015; Das and Kurita, 2020) and lower extremity (Ding et al., 2014). The SRWR devices for wrist generate flexion/extension, radial/ulnar deviation and supination/pronation motions from pneumatic soft actuators. The orthosis for wrist rehabilitation in Bartlett et al. (2015) consists of crossing linear McKibben actuators on both the palmar and dorsal sides of the forearm, providing assistance in 3 degree-of-freedom (DoF) motions. A 4-PAM actuated 2-DoF exoskeletal wrist (EXOWRIST) was presented in Andrikopoulos et al. (2015). A wrist rehabilitation exoskeleton robot actuated by 5 PAMs, including extended bending and contraction muscles, was proposed in Al-Fahaam et al. (2016) generating 2-DoF motions. A flexible wearable wrist power glove (Yao et al., 2018) was designed and manufactured utilizing four contraction PAMs at both sides of the wrist, achieving 2-DoF wrist rehabilitation motions. A soft robotic wrist sleeve actuated by two 3D printed pneumatic actuators was proposed in Ang and Yeow (2019), achieving 2-DoF motions. While the motions of the wrist were achieved, the profiles of the state-of-the-art SRWR devices were significantly enlarged by the size of the existing soft pneumatic actuators, creating difficulty when putting on and discomfort when moving repetitively. The bulky profile and the wearing complexity hindered the application in daily use.

In this work, a compact soft robotic (SR) wrist brace has been proposed. Different from the state-of-the-art SRWR wrist devices, the proposed SR wrist brace consists of eight modular soft origami actuators (SOAs), a commercially available wrist brace, and rigid anchors made of fabric. With a synergistic actuation approach, the extension/flexion and radial/ulnar deviation of the wrist are generated. Superior features in low profile, compactness, range of motion, and light weight have been achieved. The main contributions of this work are summarized as follows:

1) Proposing a modular soft actuator approach for composing wearable robotic devices. Each actuator module could be identical, while the end product can be customized to exclusively accommodate individual user through simple composition of modules.
2) Proposing a synergistic actuation approach utilizing the contraction and extension of the SOAs to realize bidirectional human revolute joint actuation, such that the actuation of joints could achieve large range of motion, under a low-profile, compact-sized form. Due to the folding and unfolding of the predefined Yoshimura pattern, the SOAs can contract and elongate from their neutral state (1 atm) when depressurized (<1 atm) and pressurized (>1 atm), respectively. This is different from the existing SRWR wrist device presented in Andrikopoulos et al. (2015) that achieved collaborative-antagonistic actuation by initially inflating the McKibben actuators at the half of the stroke (>1 atm), so that the actuators can elongate and contract.
3) Developing a soft robotic wrist actuation device using soft and rigid components, including a commercially available wrist brace and mass-fabricated SOAs through blow molding. The repeatability of the SOAs was proved by mechanical experiments. Rigid anchors, made of fabric and solidified by EVA glue, were used to install the actuators and transmit force.

The paper is organized as follows. The concept, design, fabrication, actuation, and control of the soft wearable brace were presented in *Materials and Methods*. The experiments on the functions of the brace were described in *Results*, along with the analysis on the test results. *Discussion* discusses and concludes the work with future works presented.

## Materials and Methods

The SRWR wrist devices have showed promising application potentials. To obtain compactness, low profile, and light weight, a mass-production-ready SR wrist brace is proposed in this work.

### Concept of the Soft Robotic Wrist Brace

**FIGURE 1:**
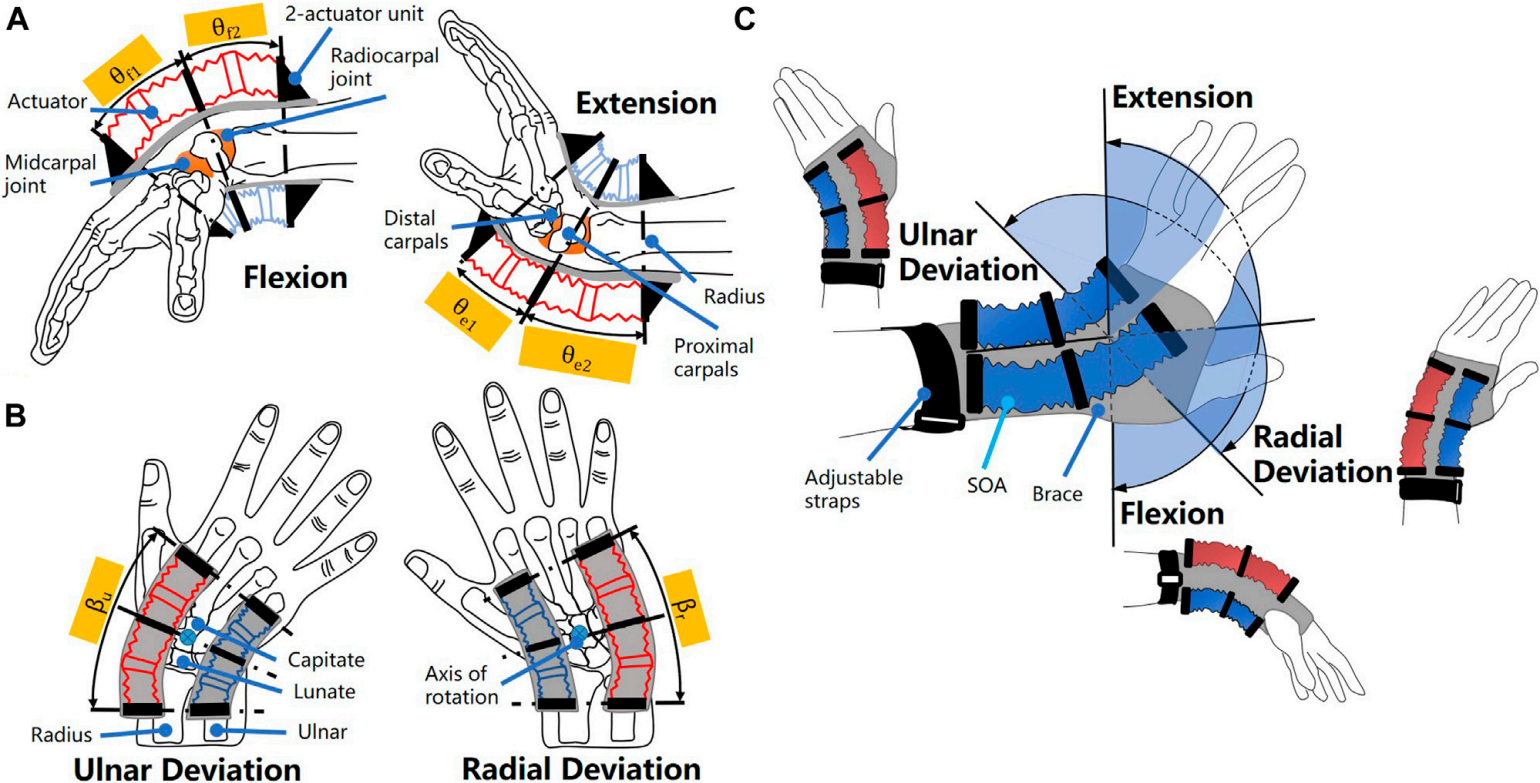
Concept of the SR wrist brace. The anatomy and arthrokinematics of the wrist in **(A)** flexion/extension and in **(B)** ulnar/radial deviation; **(C)** the definition of the 2-DoF wrist motions.

To obtain a compact design of the wearable wrist device that moves the joint in 2-DoF rotation, i.e., extension/flexion and ulnar/radial deviation, the anatomy and arthrokinematics of the human wrist were analyzed as shown in Figure 1. The wrist motion in flexion and extension was studied in Sarrafian et al. (1977). At the skeleton level, the wrist joint is comprised of two consecutive joints, the midcarpal and the radiocarpal joint, which rotate simultaneously during extension and flexion. The midcarpal joint connects the distal and proximal carpals, namely, the lunate and capitate, while the radiocarpal joint locates between the carpals and radius. Based on the average measurements, during flexion of the wrist, 40% of the motion takes place at the radiocarpal joint *θ*_f2_ and 60% occurs at the midcarpal joint *θ*_f1_. Wrist extension is measured 66.5% at the radiocarpal joint *θ*_e2_ and 33.5% at midcarpal *θ*_e1_ (see Figure 1A).

The ulnar and radial deviations occur around an axis that passed through the capitate (Ombregt, 2013), as shown in Figure 1B. During ulnar deviation, the scaphoid/lunate roll toward ulna and glide toward radius; in radial deviation, scaphoid/lunate roll toward radius and glide toward ulna.

According to Palmer et al. (1985), the functional range of wrist motion is between 5° of flexion (*θ*_f_ = 5°), 30° of extension (*θ*_e_ = 30°), 10° of radial deviation (*β*_r_ = 10°), and 15° of ulnar deviation (*β*_u_ = 15°), when performing standardized tasks. To generate the functional range of wrist joint (*θ*_f_ = 5°, *θ*_e_ = 30°, *β*_r_ = 10°, *β*_u_ = 15°) while obtaining compactness and comfortable wearing experience, a soft robotic wrist brace was proposed as shown in Figure 1A.

Soft actuators were tightly attached to the brace around the wrist in a parallel arrangement. To enhance the bending force applied on the wrist and reduce the length of the active section, the synergistic actuation approach was chosen to control the actuators in pairs. Thus, the actuators at the dorsal side of the wrist elongate while the actuators at the palmar side contract during the flexion, and vice versa during extension. The actuators in elongation and contraction state are marked as red and blue, respectively, as shown in Figure 1A. Similarly, the actuators at the radial side extend while the ones at the ulnar side contract during ulnar deviation as shown in Figure 1B, and vice versa during radial deviation.

In order to accommodate the discretized flexion/extension at the midcarpal and the radiocarpal joints, two actuators were connected into a unit with each actuator placed corresponding to one of the consecutive joints, as shown in Figure 1A. To obtain the ulnar/radial deviation, two units of actuators should be placed parallelly at the dorsal/palmar side of the wrist, with the axis of rotation located in between as shown in Figure 1B. Thus, four units of actuators in total were implemented to obtain 2-DoF wrist motions. To further reduce the complexity of the device, eight actuators were designed to be identical modules.

To apply the actuation force to the user wrist, a commercially available wrist brace was used to mount the units of actuators. The ends of the actuators were fixed on the anchors, which were then attached to the brace as shown in Figure 1C. To obtain a comfortable wearing sensation, the anchors were made of fabric materials. In one unit, there are three anchors with two actuators fixed in between. Using this arrangement, the soft robotic brace can be customized to perfectly fit a variety of wrist with different dimensions; hence the adaptivity of the SR brace can be achieved.

To keep the simplicity of the actuation module, the soft actuator was designated to output linear deformation and force in axial direction. To generate 2-DoF wrist rotations, the linear output of the actuators was mechanically reprogramed into bending, using the fabric of the brace as constrain. Due to the compliance of the soft actuator, the bending output was generated.

### Design and Fabrication of SOA

To obtain a light-weight and soft wearable device, soft pneumatic actuators were chosen to construct the brace. In recent years, the soft origami actuators have gained popularity due to their advantages in output force, efficiency, and programmable movements (Martinez et al., 2012; Paez et al., 2016; Li et al., 2017). The innovations in the design of origami patterns have boosted the potential of the soft origami robotic systems in new applications (Liu et al., 2016; Yi et al., 2018; Zhang and Chen, 2019). However, these actuators assembled at least two components to generate desired movement, using the origami mechanism as added reinforcement or constrain to the isotropic expanding air cavity. The complexity in the fabrication of actuator hinders the repeatability in mechanical performance, especially in mass production.

**FIGURE 2:**
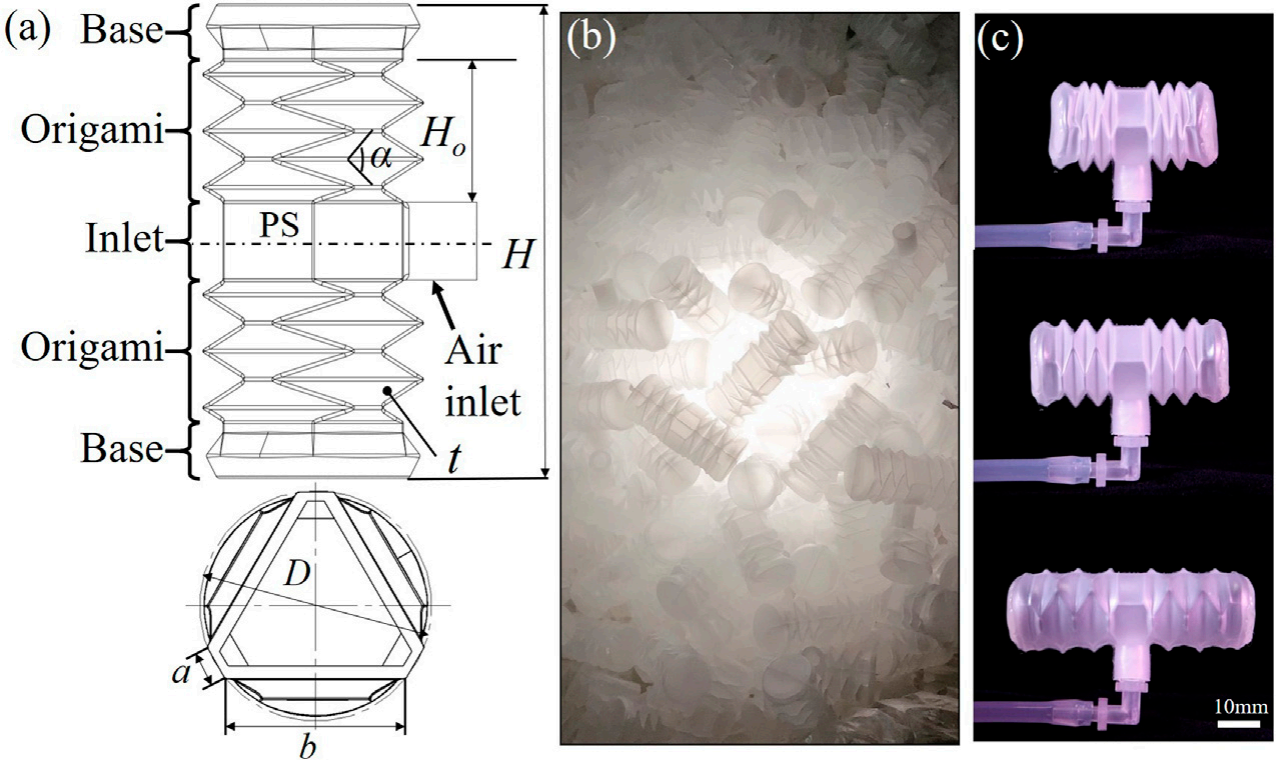
Design of soft origami actuator. **(A)** The definition of parameters; **(B)** a batch of 500 units were produced by a blow molding run; **(C)** the actuator contracts and elongates when depressurized and pressurized, respectively.

To design the soft actuator with simplified structure and mass-production potential, a variation of Yoshimura pattern with identical hexagonal facets was adopted and directly implemented on the walls of air cavity as in Guo et al. (2019); see Figure 2. The Yoshimura pattern has shown promising potential as the expansion actuators, due to its small elastic constant in the axial direction, simplicity of elementary facets, and intrinsically high circumferential bending rigidity (Miura, 1969). Thus, the Yoshimura pattern can be applied on a pneumatic soft actuator obtaining deformation in the axial direction like the bellows, while attaining superior stability in the circumferential direction. The hexagon facets effectively eliminate the extruding vertices formed by the triangular facets of the standard Yoshimura pattern; thus, the potential stress concentration at the vertices was avoided.

As shown in Figure 2A, the soft origami actuator (SOA) consists of three functional sections, i.e., the base, origami, and inlet. The origami sections generate the axial elongation and contraction movement when pressurized and depressurized, respectively. The base sections were used to mount on the anchors and to apply force to contacting surfaces. The origami and base sections were arranged symmetrically to the plane of symmetry (PS). For an easy assembly process, the air inlet was placed at the middle section of the actuator in between the origami sections, achieving maximum contacting area at both base sections. The dimensions of the SOA were defined in Figure 2A and listed in Table 1.

**TABLE 1:** Geometry of the SOA.

*H*	Height of SOA	42 mm
*H*_*o*_	Height of the origami section	13 mm
*t*	Thickness of origami facet	0.5 mm
*α*	Dihedral angle of two trapezoid facets	65.6°
*D*	Diameter of the circumcircle	20 mm
*a*	Length of short parallel side of facet	2.9 mm
*b*	Length of long parallel side of facet	15.1 mm

In order to produce the SOAs in large amount with low cost and high production rate, the industrial mass-production process blow molding was chosen. The highly commercialized material ethylene-vinyl acetate copolymer (EVA) by FORMOSA was used to make the SOAs, in consideration of its compliance, availability, cost, and biocompatibility. The fabricated SOAs were presented in Figure 2B. A batch of 500 pieces were made in one production run. The single SOA weighs around 1.8 g with thickness of 0.5 mm. As shown in Figure 2C, the maximum contraction and elongation of the SOA were measured as 30 mm at 70 kPa and 55 mm at 185 kPa, respectively.

An analytical model of the SOA has been derived in Su et al. (2020); the relationship of the output force *F*, axial deformation ΔH, and the relative pressure in the air cavity $\Delta P_{a}$ is given as follows:

$$F=F_{k}\left(\Delta H\right)+F_{p}\left(\Delta P_{a}\right),$$ (1)

where $F_{k}\left(\Delta H\right)=k\Delta H,$ is the force generated by the elastic deformation of the SOA, and k is the equivalent coefficient of elasticity; $F_{p}\left(\Delta P_{a}\right)=S\Delta P_{a},$ is induced by the relative pressure in the air cavity of SOA, and S is the effective area of the actuator subject to inner pressure.

### Fabric Constraint on 2‐SOA Unit

The axial deformation and output force of the SOA were predefined by the origami pattern. To apply on the wrist brace and move the joint in 2-DoF revolutions, the output of the SOAs should be reconfigured to bending, in a way that the lateral side of the SOAs conforms to the wrist curvature and one end of the SOA rotates from the other, as shown in Figure 1. This requires anisotropic deformations when the SOA is pressurized and depressurized; i.e., the proximal side of the SOA deforms less than the distal side.

**FIGURE 3:**
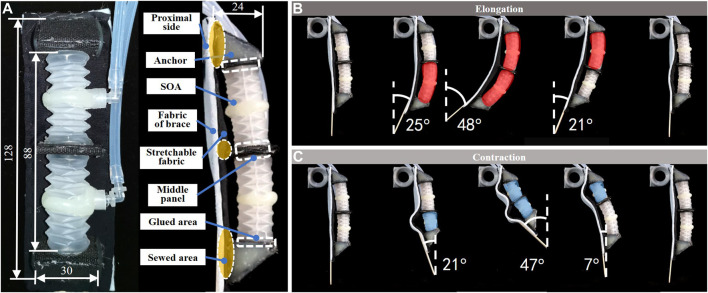
A 2-SOA unit, **(A)** the unit was made of soft materials; the axial deformations of SOAs were constrained by brace fabric; **(B)** when pressurized, the bending angle reached 48°; **(C)** when depressurized, the bending angle reached 47°.

To generate bending from the SOA, the fabric of the brace was used to reconfigure the linear deformation by constraining the proximal side of the SOAs, as shown in Figure 3A. The approach to reprogram the deformation of soft actuators by added fabric stripe has been presented (Martinez et al., 2012; Connolly et al., 2019). In this work, the fabric constraint was applied on multiple actuators in a series arrangement, which generated multiple DoFs of bending, while in prior work the fabric constrain was applied on single air cavity which generated 1 DoF of bending.

Aiming for stable force-transmission links, nonstretchable nylon fabric was used to fabricate the SOA anchors. The nylon fabric stripes were cut, folded, and glued into rigid pentahedron anchors and flat middle panels. The dimensions are shown in Figure 3A. Spandex fabric with exceptional elasticity was used as the intermediate layer to connect the anchors, before being installed onto the brace. The spandex layer was glued to the pentahedron anchors and middle panel.

2-SOAs were installed in between the anchors for an adequate range of motion, by gluing the base sections to the nylon fabric. Then, the spandex layer along with the installed actuators was sewed to the fabric of brace. For a reliable force-transmission route. The sewed areas include the periphery of the spandex, the contacting areas of the pentahedron, and middle panel. The glued and sewed areas are displayed in Figure 3A.

The bending deformation of the 2-SOA unit was shown in Figures 3B,C. The two SOAs were individually controlled to show mobilities and range of motions. The activated SOAs were marked red and blue corresponding to elongation and contraction, respectively. When pressurized as in Figure 3A, the SOAs rotated around the constrained side, with maximum rotation of 48°. When depressurized as in Figure 3C, the contraction of the SOAs was obstructed by the fabric; thus, the unit bent around a virtual axis at the distal side. The maximum rotation during contraction was around 47°. The fabric of brace is considerably thicker (2.5 mm) than the spandex layer (0.3 mm), such that the constrains applied on the deformations of SOAs are generated by the fabric of brace.

### Design and Fabrication of Soft Robotic Brace

**FIGURE 4:**
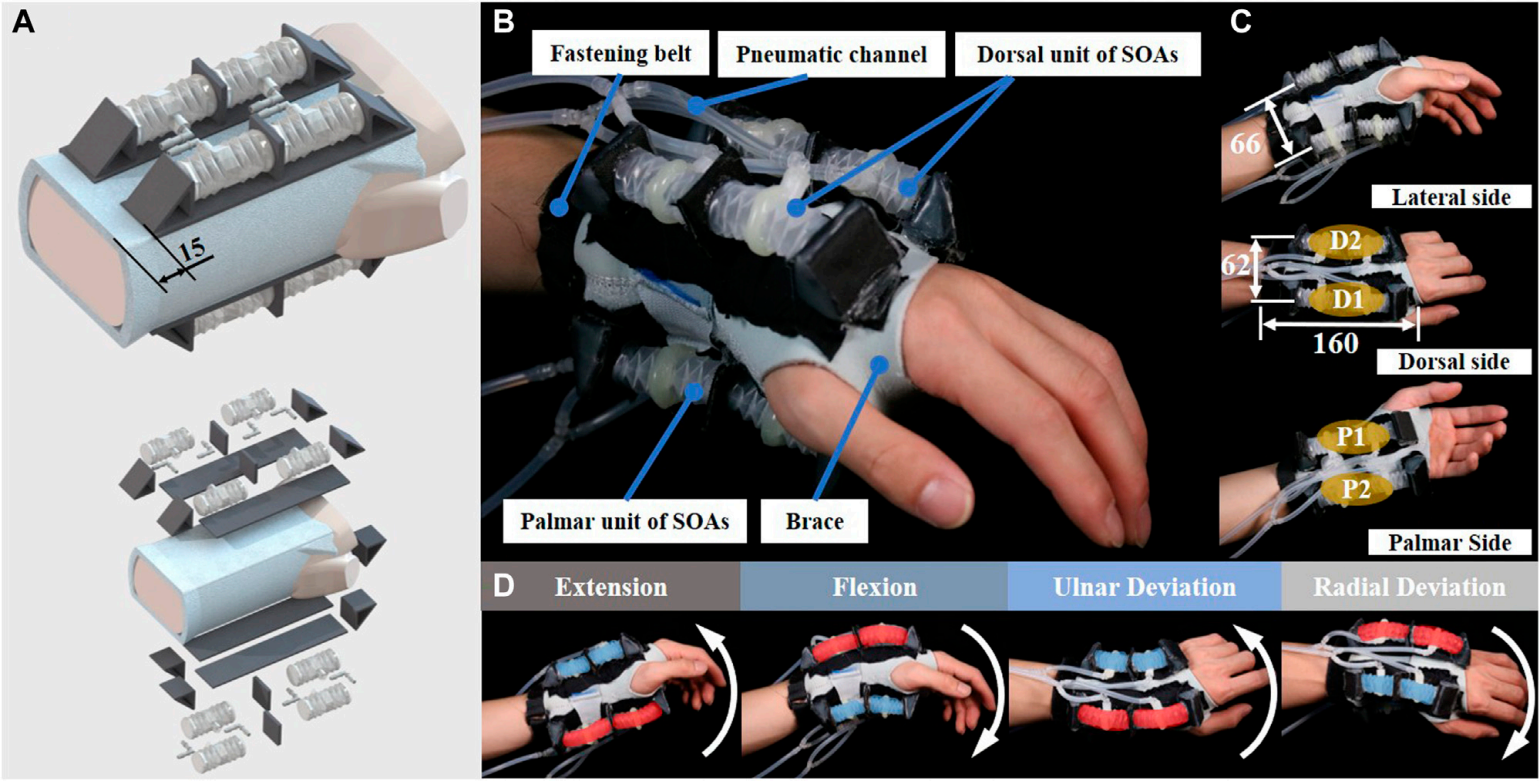
Design of SR brace and the prototype, **(A)** the soft robotic brace incorporates eight SOAs into four 2-SOA units at the palmar and distal side of the wrist; **(B)** the SR brace prototype worn on human wrist; **(C)** the dimensions of the brace; **(D)** the brace moved the human wrist in flexion, extension, ulnar, and radial deviation.

The design of the soft robotic wrist brace consists of four identical 2-SOA units and a brace is shown in Figure 4A. To obtain 2-DoF wrist motions with a low-profile form, the 2-SOA units were tightly sewed on the commercially available brace. A two-by-two parallel arrangement of the 2-SOA units was adopted, namely, two units at the dorsal side and two at the palmar side. The locations of the units were determined by the axes of joints. As mentioned in *Concept of the Soft Robotic Wrist Brace*, the midcarpal and radiocarpal joints rotate simultaneously during flexion and extension; thus the units were placed with the middle panels located at the proximal carpals, as shown in Figure 1. During the ulnar/radial deviations, the axis of rotation was considered passing through the capitate; thus the virtual plane coplanar with middle panels should pass through capitate.

The fabricated soft robotic brace was worn and shown in Figure 4B. In order to maximize the torque applied on the wrist while considering the range of motion, the locations of the 4 units are determined as shown in Figure 4C. The distance between two units was listed. As shown in Figure 4B, a hook-and-loop fastener belt was sewed on the proximal end of the brace to prevent slipperiness on the forearm. The weight of the soft robotic wrist brace worn on the user is around 241 g, which is comparable to the state-of-the-art light-weight wrist device (Al-Fahaam et al., 2016; Bartlett et al., 2015), while the profile is significantly smaller (Andrikopoulos et al., 2015; Bartlett et al., 2015; Al-Fahaam et al., 2016; Ang and Yeow, 2019). The dimensions of existing soft robotic wrist devices and actuators are listed and compared in Table 2. The proposed SR wrist brace only covers the palm and wrist areas of the user, due to the use of short modular SOAs, while state-of-the-art devices (Andrikopoulos et al., 2015; Bartlett et al., 2015; Yao et al., 2018; Ang and Yeow, 2019) include palm, wrist, forearm, or even the elbow to install longer actuators. Thus, our proposed SR wrist brace achieved compactness in terms of the wearing area on user’s arm.

**TABLE 2:** Comparison with the existing soft robotic wrist wearable devices.

SR wrist device	Length (mm)	Muscle length (mm)	DoF	Weight (g)	Wearing position
EXOWRIST Andrikopoulos et al. (2015)	>214	PAM: 214	2	430	Palm and forearm
Soft rehab. exoskeleton Al-Fahaam et al. (2016)	>200	Contraction muscle: 200	2	150	Palm and wrist
Flexible wearable wrist power glove Yao et al. (2018)	>160	PAM: 160	2	337	Palm and forearm
3D printed soft robotic wrist sleeve Ang and Yeow (2019)	—	—	2	—	Palm and forearm
Soft robotic orthosis for wrist rehab Bartlett et al. (2015)	—	—	3	220	Palm and elbow
ASSIST Sasaki et al. (2005)	245	Soft actuator: 180	1	390	Palm and wrist
Proposed SR brace	160	2-SOA unit: 128	2	241	Palm and wrist

The movement of the soft robotic brace worn on the user’s wrist is shown in Figure 4D. The two SOAs in each unit were connected to the same fluid channel; thus each 2-SOA unit has 1 DoF. Therefore, the SR brace has four independently controlled actuation units. In order to enhance the torque and range of motion, the units D1 and D2 are synchronized during flexion/extension, while P1 and P2 are synchronized. During ulnar/radial deviation, the D1 and P1 are synchronized, while D2 and P2 move in the same direction. Thus, the two synchronized pairs move the wrist in a synergistic fashion. The SR brace moved the user’s wrist in extension, flexion, ulnar, and radial deviation as shown in Figure 4D, with the elongating units marked red and the contracting ones marked blue.

### Actuation and Control

**FIGURE 5:**
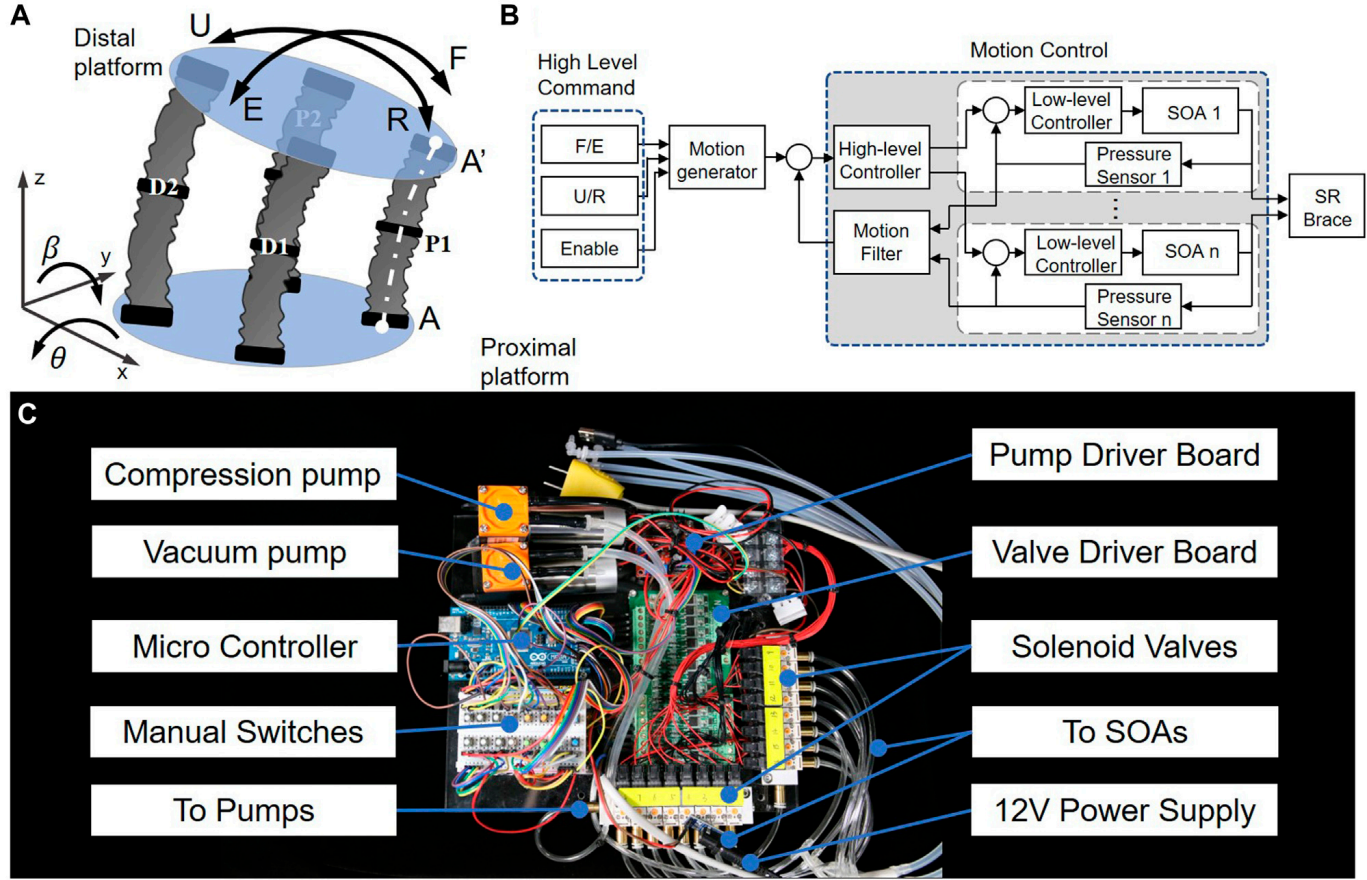
The principle of actuation and control, **(A)** the brace can be considered as a parallel mechanism actuated by soft actuators; **(B)** a cascaded control strategy was used; **(C)** the dimensions of the actuation system are 24 cm*23 cm*6 cm and the weight is 1.76 kg.

The mechanism of the SR brace can be simplified into a parallel mechanism with four soft actuators, as shown in Figure 5A. The distal platform moves in 2-DoF rotations relative to the fixed proximal platform. The datum and coordinate system are defined according to the proximal platform. Thus, the flexion and the extension of the distal platform are defined as the rotation around the *x*-axis, while the ulnar and radial deviation are defined as the rotation around the *y*-axis. Point A denotes the intersection point of the axis of the unit P1, while A’ denotes the intersection point of the axis and the distal platform.

The position of point A’$\;\left(x_{a}^{\prime },y_{a}^{\prime },z_{a}^{\prime }\right)$ can be obtained from the position of point A $\;\left(x_{a},y_{a},z_{a}\right)$, by angular displacements θ and β around the *x*-axis and *y*-axis, respectively, and the translation $z_{0}$ along the *z*-axis. Thus, according to the Denavit-Hartenberg matrix, the following relationship can be derived:

$$\left[\begin{array}{c} x_{a}^{\prime }\\ y_{a}^{\prime }\\ z_{a}^{\prime } \end{array}\right]=\left[\begin{array}{ccc} \cos\beta & 0 & \sin\beta \\ 0 & 1 & 0\\ -\sin\beta & 0 & \cos\beta \end{array}\right]\left[\begin{array}{ccc} 1 & 0 & 0\\ 0 & \cos\theta & -\sin\theta \\ 0 & \sin\theta & \cos\theta \end{array}\right]\left[\begin{array}{c} x_{a}\\ y_{a}\\ z_{a} \end{array}\right]+\left[\begin{array}{c} 0\\ 0\\ z_{0} \end{array}\right],$$ (2)

where $z_{a}=0$; thus, the position of A’ can be simplified as

$$\left[\begin{array}{c} x_{a}^{\prime }\\ y_{a}^{\prime }\\ z_{a}^{\prime } \end{array}\right]=\left[\begin{array}{l} \cos\beta x_{a}+\sin\beta \sin\theta y_{a}\\ \cos\theta y_{a}\\ -\sin\beta x_{a}+\cos\beta \sin\theta y_{a}+z_{0} \end{array}\right].$$ (3)

Thus, the distance between A and A’, i.e., $H_{P1}$, the length of the unit P1, is derived as follows:

$$H_{P1}=\sqrt{{\left(\cos\beta x_{a}+\sin\beta \sin\theta y_{a}-x_{a}\right)}^{2}+{\left(\cos\theta y_{a}-y_{a}\right)}^{2}+{\left(z_{0}-\sin\beta x_{a}+\cos\beta \sin\theta y_{a}\right)}^{2}}.$$ (4)

When performing pure flexion or extension, β=0, the length of unit P1 is derived as

$$H_{P1}=z_{0}+\sin\theta y_{a}.$$ (5)

When performing pure ulnar or radial deviation, i.e., θ=0,

$$H_{P1}=\sqrt{{\left(\cos\beta x_{a}-x_{a}\right)}^{2}+{\left(z_{0}-\sin\beta x_{a}\right)}^{2}}.$$ (6)

According to the relationship between the pressure and the displacement of the actuator in the equation (Krakauer, 2005), the corresponding inner pressure of the P1 can be calculated when there is no loading on the distal platform. The lengths and inner pressures of the P2, D1, and D2 units can be calculated in such way. Therefore, the movement of the brace can be controlled by the pneumatic inputs.

The principle of the control for the SR brace is shown in Figure 5B, which was well documented in Zhou et al. (2019). A cascaded control structure was adopted, with a motion-control outer loop and multichannel pressure-control inner loops for each SOA. The outer loop, controlled by the high-level controller, used pressure feedback information from each actuator to obtain the overall device motion estimation and then compared with the desired motion mapped from the different control commands (flexion/extension, ulnar/radial deviation, and engagement signal). The resultant pressure commands from the high-level controller were then relayed to the low-level controllers, each regulating one soft origami actuator accordingly.

The actuation system is shown in Figure 5C. Two diaphragm pumps (Kamoer KVP8, 12 V 9 W) supply compressed air and vacuum to the actuators via eight solenoid miniature pneumatic valves (OST Solenoid SY3/2NC, 12 V 1 W). Power was supplied via external battery or 12 V DC adaptors. A microcontroller (Arduino Mega) embeds the control algorithms, with eight manual switches for manual overriding and individual actuator control. The geometric dimensions of the actuation system are 24 cm*23 cm*6 cm; the weight is measured to be 1.76 kg.

## Results

### Test on the SOA

**FIGURE 6:**
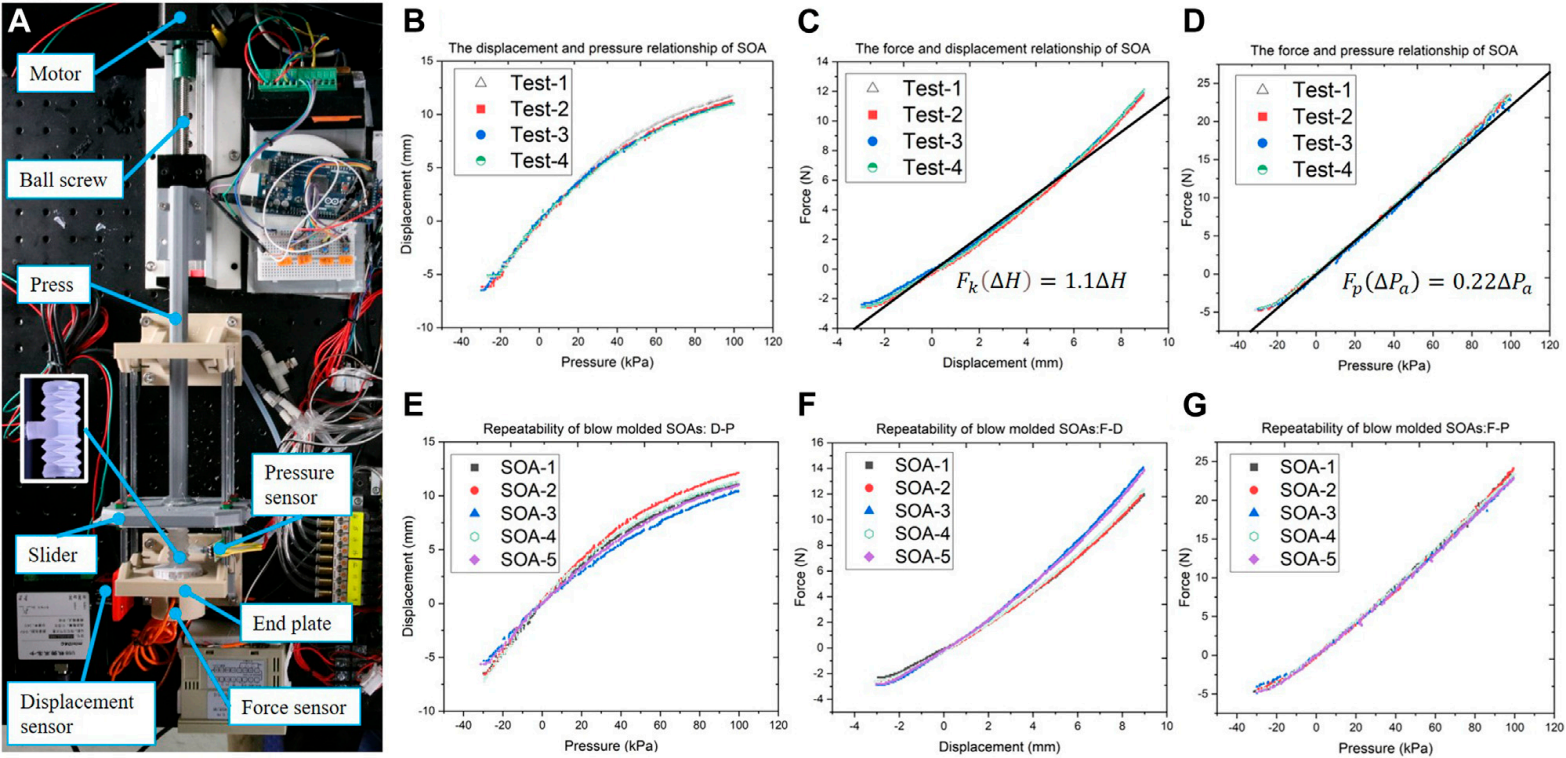
Tests on the soft origami actuator, **(A)** the test setup that measures the displacement, air pressure, and force; the test results of single SOA: **(B)** the displacement–pressure relationship, **(C)** the force–displacement relationship, **(D)** the force–pressure relationship; the repeatability tests on five SOAs of 1.9 g weight: **(E)** the displacement–pressure relationship, **(F)** the force–displacement relationship, **(G)** the force–pressure relationship.

To measure the inner pressure, the displacement, and force during the deformation of the SOA, the test setup is shown in Figure 6A. The SOAs of 1.9 g weight were mounted between the end plate and the slider. The end plate is screwed to the test platform, and the slider moves in the normal direction of the end plate collinear with the axial deformation of the SOA during test. A force sensor with range 0–50 N (DYMH-103, 5 kg, Daysensor) was placed between the SOA and the end plate. The press can be linearly motivated by the step motor (6,400 steps per revolutions) through ball screw. In order to minimize the friction of the test setup, CMOS type Micro Laser Distance Sensor (Panasonic HG-C1050, 50 mm, 30 μm resolution) was used to record the linear displacement of the slider. The pneumatic fittings connect the SOA, the pump, and the air pressure sensor (SSCDANN030PAAA5, 206 kPa, Digi-Key). During tests, the air pressure, the location of the press, and the output force were recorded.

The experiments on the mechanical properties of single SOA were carried out, with the results plotted in Figures 6B–D. The force–displacement relationship was plotted in Figure 6C, where the SOA was passively compressed by external forces. The curve was fitted by the model in the equation (Krakauer, 2005), which derived the equivalent coefficient of elasticity k=1.1. The force–pressure relationship was measured and plotted in Figure 6D, by limiting the linear deformation of the SOA at neutral length of 42 mm. The results were fitted by the model in the equation (Krakauer, 2005), which derived the effective area of the SOA S=0.22.

To study the repeatability of blow molding, which is a mass-production fabrication process, five SOAs of approximately 1.9 g weight were tested using the same setup. The results are plotted in Figure 6E–G; the force–pressure curves in Figure 6G showed high conformity. The disparities shown in Figures 6E,F increase with the deformation of the actuators, which might be induced by the differences in the thickness of origami facets among tested SOAs. Due to the principle of the blow molding process, the thickness of the origami facets cannot be precisely controlled, which limited the repeatability of the resultant SOAs. Although the repeatability of the blow molded SOAs is not as consistent as the electric motors, they showed considerate advantage over the soft actuators made of hyperelastic material by injection molding or casting. Considering the production rate and cost per unit, the blow molding process has promising potential in the mass production of soft robotic devices.

When compared with the rigid robotic actuation such as motors, the SOA has advantages in compliance, light weight, small bulk, and low cost, which benefit the wrist brace to achieve safe human-robot interaction, low profile, adaptivity, and affordability.

In comparison with the state-of-the-art soft actuators, as listed in Table 2, the SOAs have advantages in cost, weight (1.9 g), linear mechanical behavior, and mass-production readiness. The state-of-the-art McKibben actuators (Andrikopoulos et al., 2015; Al-Fahaam et al., 2016; Yao et al., 2018) suffer from force loss and hysteresis due to frictions between weave and inner tube and within weave itself (Tondu and Lopez, 1997). They possess minor pushing force during depressurization, highly nonlinear attributes that create difficulties in modeling and control (Andrikopoulos et al., 2015). Unlike the McKibben actuators, the SOA adopts the folding/unfolding motion of origami pattern to generate contraction/extension with no friction generated, which eliminates the energy loss by friction. According to the test results shown in Figure 6, the SOA displayed linear mechanical behavior that benefits the simple modeling and control of the wearable robot. The 3D printed actuator in Ang and Yeow (2019) sacrifices the compactness for the range of motion and torque, while the small dimensions of the SOAs and the modular approach grant the proposed wrist brace low profile and compactness.

### Test on the Brace

**FIGURE 7:**
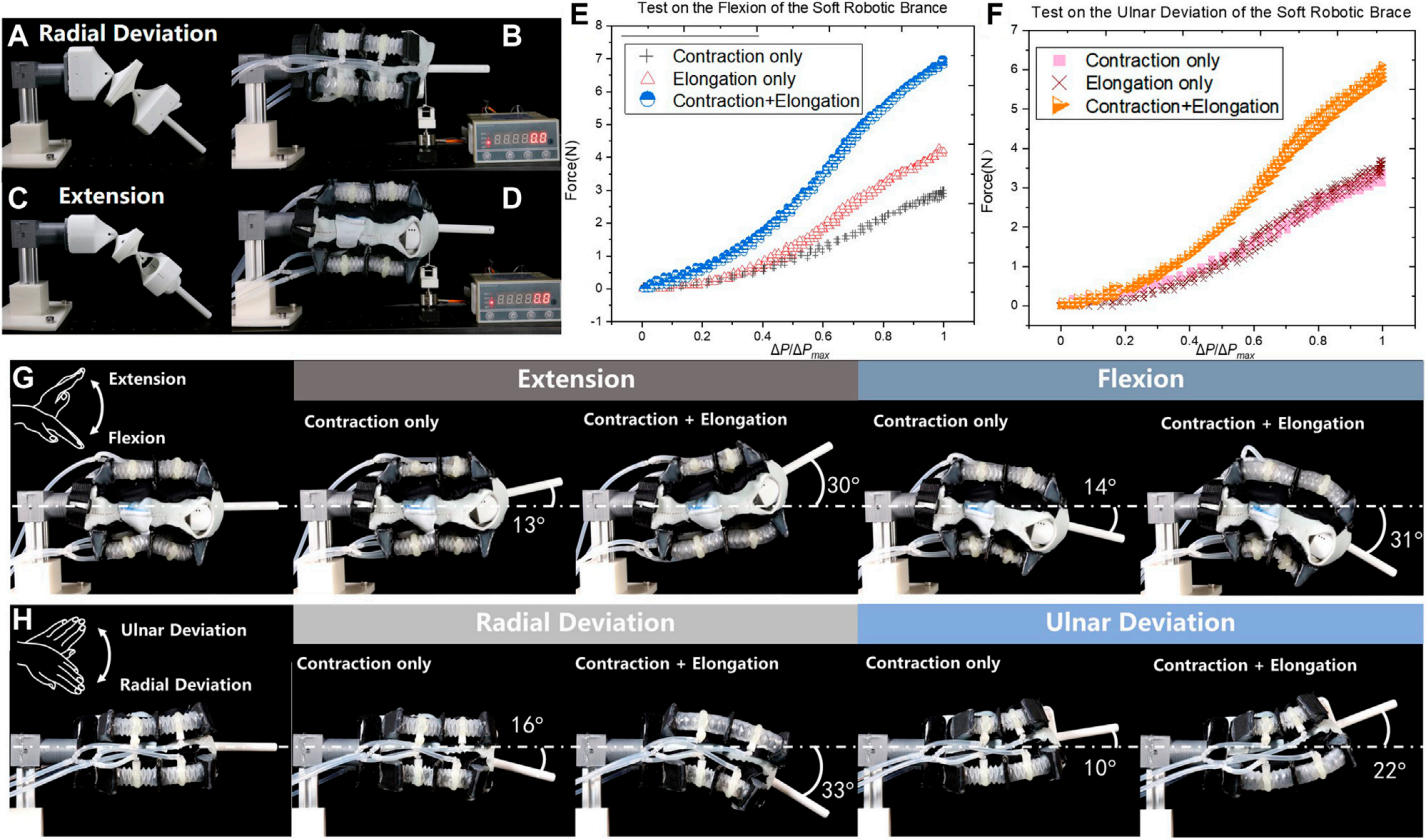
Tests on the brace, **(A)** the test rig for **(B)** the output force test in radial deviation; **(C)** the setup for **(D)** the output force test in extension, the results of the tests were plotted in **(E)** and **(F)**; the ranges of motions using contraction-only and synergistic actuation approach were compared in **(G)** extension/flexion and **(H)** radial/ulnar deviation.

The tests on the SR brace were carried out to study on the mechanical performance of the SR brace, as shown in Figure 7. The output force and range of motion were tested on the setup shown in Figure 7A–D. The SR brace was worn on the passive linkage consisting of two ball joints. The two positions of the passive linkage corresponding to the radial deviation and extension are shown in Figure 7A,C, respectively. Oval shaped links supported the brace, preventing collapsing, while the ball joints allowed the SR brace to perform 2-DoF wrist motions.

The tests were carried out in two directions according to the 2-DoF movement as shown in Figure 7B,D. Due to the symmetric building of the brace, only the radial deviation and extension at the neutral position were tested. The distal end of the brace was connected to the force sensor by a nonstretchable thread.

The results of the flexion tests are plotted in Figure 7E and the ulnar deviation in Figure 7F, where the relationships between the output force F and the normalized pressure input $\Delta P/\Delta P_{\max}$ were displayed, where ΔP is the pressure difference between the pressurized and depressurized actuators. Three driving modes, contraction-only, elongation-only, and synchronistic-contraction-elongation, were compared. According to the curves, the synchronistic approach outputs significantly higher force than the other two modes at the same normalized pressure point, while the contraction-only mode showed the least output force.

The range of motion in extension/flexion and radial/ulnar deviation was recorded and measured as shown in Figure 7G,H. The contraction-only and the synchronistic approach were activated consecutively. In four cases, the output angular displacements were significantly increased when synchronistic approach was adopted in comparison with the contraction-only mode. The maximum range of the motions is measured as extension 30°, flexion 31°, radial deviation 33°, and ulnar deviation 22°. The ulnar deviation displayed smaller angular displacement due to the loose bonding anchors in unit D2, which less effectively transmitted the contraction of D2.

**FIGURE 8:**
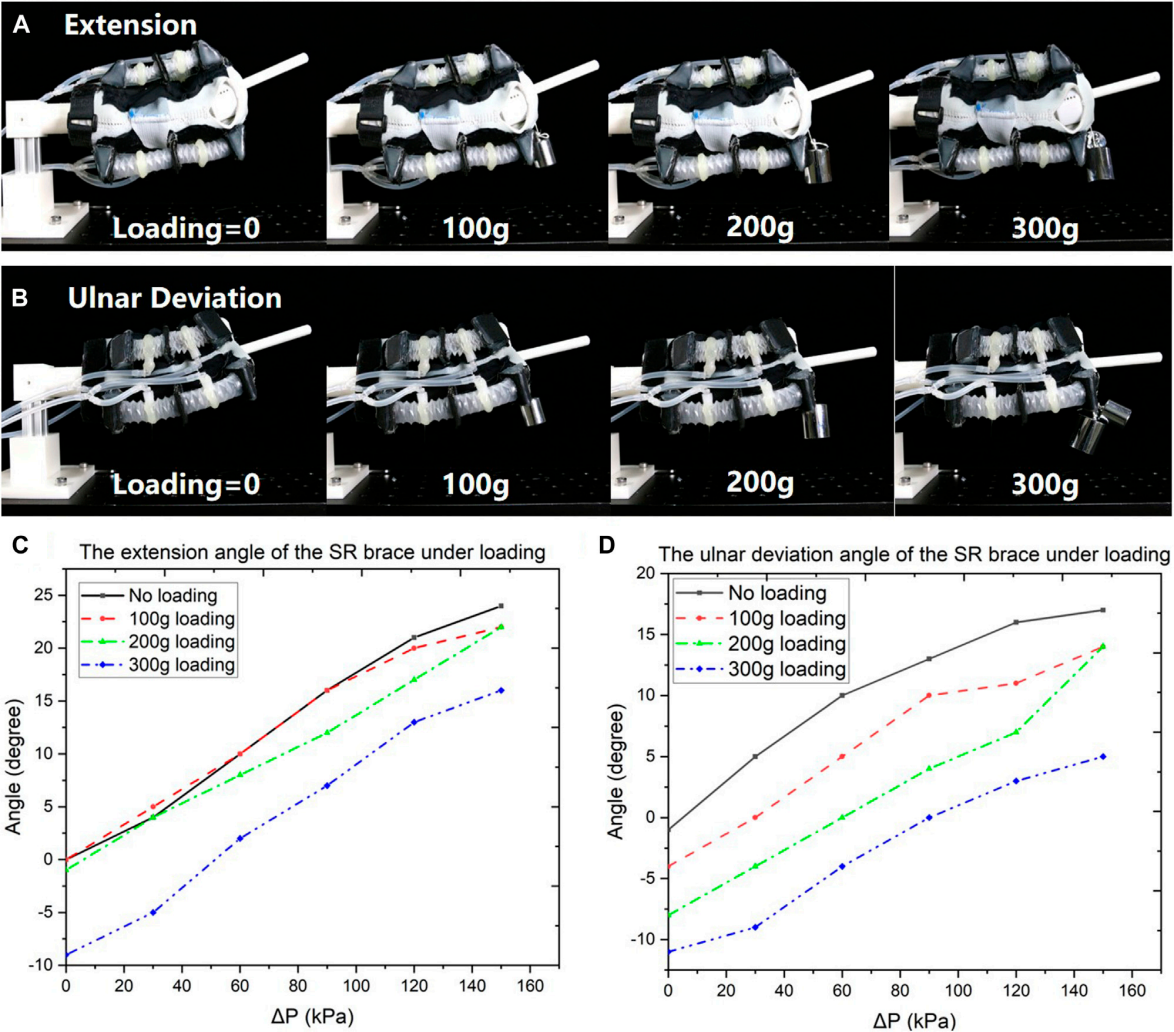
The performance of the SR brace under loading was measured. The weights 100, 200, and 300 g were attached to the distal end of the SR brace; the output angles were measured in **(A)** extension and **(B)** ulnar deviation.

To investigate the performance of the SR brace under external loading, weights of 100, 200 and 300 g were attached to the distal end of the brace and output angles were measured as shown in Figure 8. The tests of extension and ulnar deviation are shown in Figures 8A,B, respectively. The test results of the extension were plotted in Figure 8C; the curves under no loading and 100 g loading show small disparities at ΔP=145 kPa. The curve under 200 g loading shows slightly larger disparities from the no-loading curve when ΔP>90 kPa. When under 300 g loading, the output angle of SR brace deviated significantly from the no-loading curve. Thus, considering the complaint nature of the soft robotic systems, the SR brace moved close to its no-loading performance under loading smaller than 200 g during extension. The curves plotted in Figure 8D display a less resilient performance under loading during ulnar deviation. The curve under 100 g loading shows large disparities from the no-loading curve, and the disparities deteriorate with the load.

**FIGURE 9:**
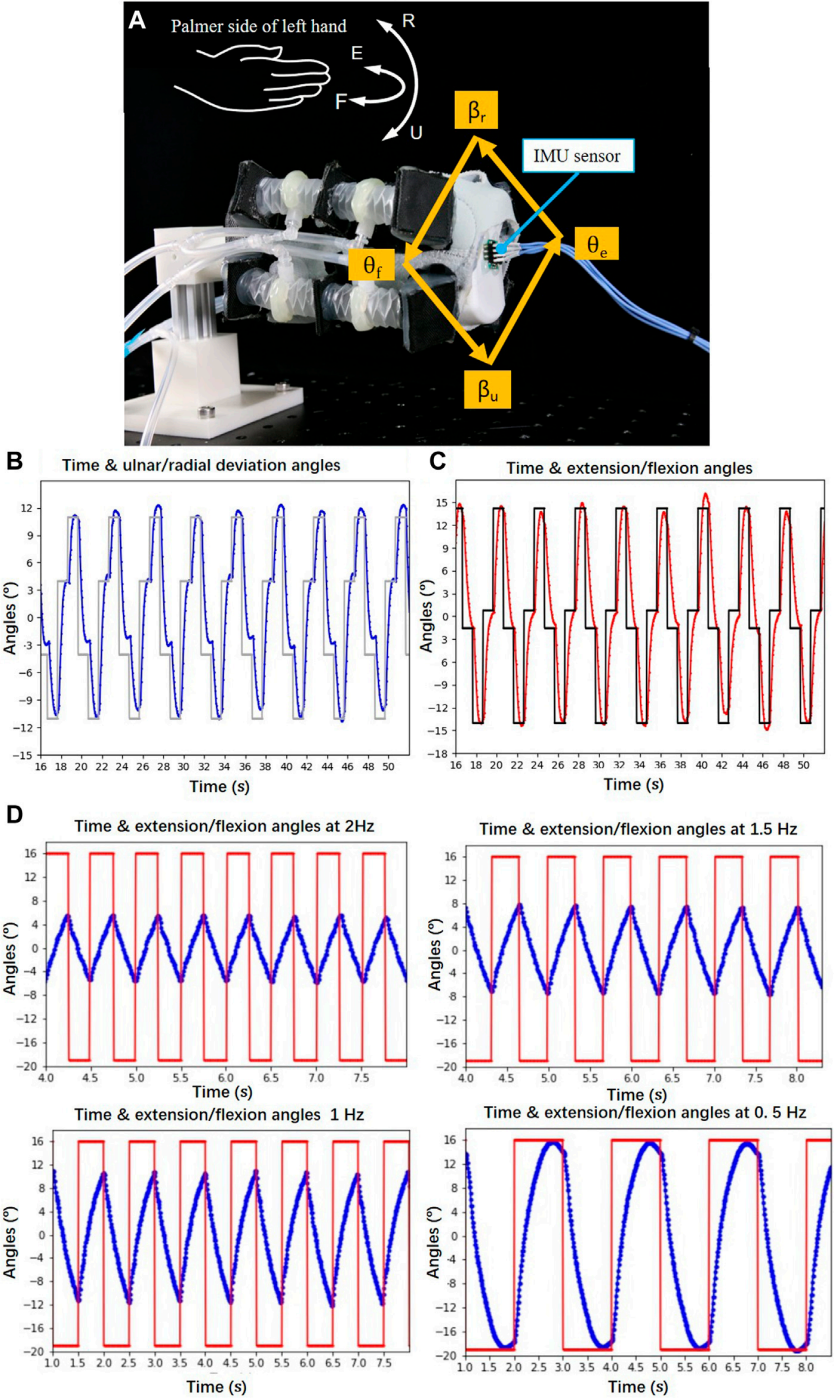
The control performance was tested; **(A)** a IMU sensor was attached to the distal end of the test rig to measure the motion of the SR brace; a quadrilateral loop route was defined for the end of the brace to follow; **(B)** the ulnar/radial deviation angles *β*_*u*_ and *β*_r_ have been plotted against time; **(C)** the extension/flexion angles *θ*_e_ and *θ*_f_ were plotted against time; **(D)** the bandwidth of the brace was studied.

The performance of the control was tested in a setup shown in Figure 9A, where a IMU sensor was attached to the distal end of the test rig to measure the motion of the SR brace. A 6-axis motion tracking sensor MPU6050 (Gyro + Accelerometer, accuracy 0.1°, WitMotion) was chosen as the IMU. As shown in Figure 9A, a quadrilateral loop route was defined for the distal end of the SR brace to follow. The loop required the SR brace to move in the extension, radial deviation, flexion, ulnar deviation sequence and repeat. The high-level controller shown in Figure 5B calculated the pressures of each actuator from the angular position set by the loop. The theoretical model of the SR brace represented by the equation (Krakauer, 2005; Celestino, 2003; Ferreira et al., 2018) was used in the high-level controller. The motions were captured and plotted in Figure 9B,C, displaying the angular displacement in the ulna/radial and extension/flexion directions, respectively. The output of the SR brace followed closely and consistently to the planned value in the extension/flexion direction, while it showed less stable behavior in the ulnar/radial direction. The different behavior might be caused by the inconsistency induced by the fabrication of the SR brace. Considering the compliant nature of the system, the performance of control is acceptable.

The bandwidth of the brace was studied using the same setup shown in Figure 9A. The brace was given periodic step reference signals ranging from *θ*_e_ = 16° to *θ*_f_ = 18° at 2, 1.5, 1, and 0.5 Hz operating frequency, respectively. The results were plotted in Figure 9D. The response of the brace showed that it can reach the reference angles at 0.5 Hz, while the range of motion was shortened at higher frequencies.

**FIGURE 10:**
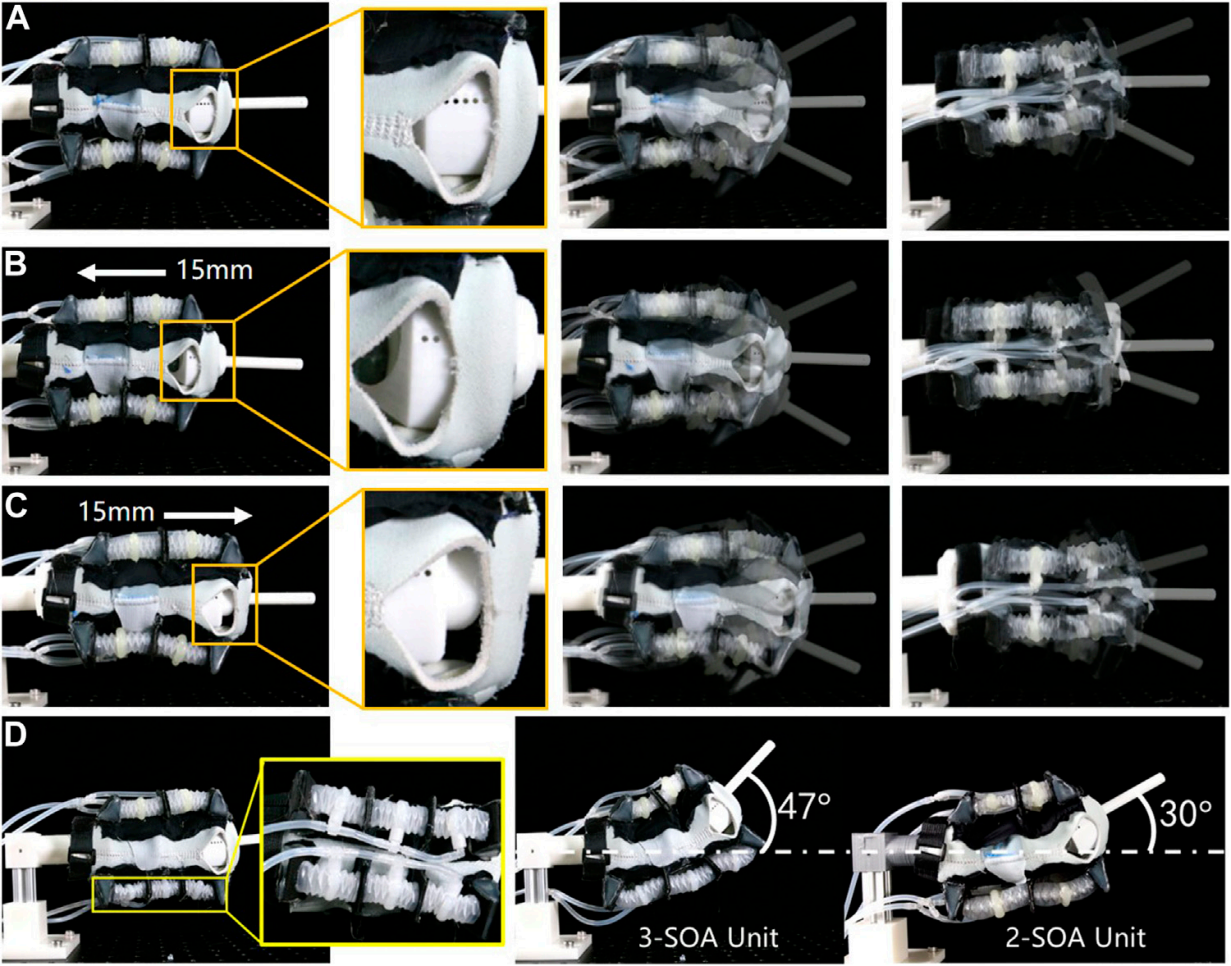
The tests on the adaptivity in wearing position and the modularity of the SOA were carried out; **(A)** the SR brace was worn on the test rig at the neutral position; **(B)** the SR brace was pulled 15 mm toward the forearm; **(C)** the SR brace was moved 15 mm toward the fingers; **(D)** by replacing the 2-SOA units with 3-SOA units at the palmar side, a new variation of the SR brace was produced with increased extension angle.

The wearing position of the SR brace on human wrist can vary according to user’s personal preference. The dimensions of the wrists are also different among the users. Thus, the wearing device should adapt to a range of sizes and wearing positions, while obtaining the motion assistive effect on the wrist joint. Due to the flexible fabric of the commercially available brace, the SR brace is inherently adaptive to various wrist sizes. To demonstrate the adaptivity in the wearing position, experiments were carried out as shown in Figures 10A–C. The position of the SR brace changed relative to the test rigs to imitate the variation of the wearing positions on human wrist. The brace was pulled toward the forearm by 15 mm in Figure 10B, compared to the position shown in Figure 10A. The brace was moved toward the fingers by 15 mm in Figure 10C. The movements were captured and compared, which showed small changes in the range of the motion in both directions. Thus, the wearing position of the SR brace does not have to be precise to achieve the motion assistive function.

To demonstrate the modularity of the actuators and the practicality of the modular approach, a new variation of SR brace was fabricated as shown in Figure 10D. The 2-SOA units at the palmar side the of the brace were replaced with 3-SOA units. The 3-SOA unit was straightforwardly fabricated by adding one SOA and one middle panel to the 2-SOA unit. The maximum extension angle of the new SR brace was then measured (*θ*_e_ = 47°), which increased considerably from the original prototype (*θ*_e_ = 30°). The result shows the potential of the modular approach in customization and application in other wearable devices.

## Discussion

Compactness and wearing comfort contribute critically to the adoption of SRWR devices. In this work, a compact, light-weight soft robotic wrist brace has been proposed. Incorporating eight SOA modules assembled in 4 units onto a commercially available fabric wrist brace, the proposed SR wrist brace prototype was tested in a series of validation experiments. Results presented have demonstrated the performances and advantages of the proposed approach, as follows.

1) The proposed modular soft actuator approach in composing wearable robotic devices was effective and led to customizable wearable robot designs. While each module actuator could be identical, the end product could be customized to fit each user through simple composition of modules. We also proposed a soft-on-soft approach to constrain and preprogram the output of the SOAs, where the extension of the SOAs was constrained by fabrics into consecutive bending. As a result, the output of actuator can be customized. The adaptability of the brace can be obtained by designing the appropriate fabric constrain according to the dimensions of the user’s wrist. Consequently, the motion of the actuator was simplified; the design of the actuator can be unified. As a result, the SOAs were fabricated using commercially available material EVA by a cost-efficient mass-production industrial process, namely, the blow molding.
2) The proposed synergistic approach to realize bidirectional human revolute joint actuation was effective in generating multi-DOF human wrist motion. The synergistic actuation of joints could achieve large bending range, under a low-profile, compact-sized form. By utilizing the elongation and contraction movement of the SOAs when pressurized and depressurized respectively, the synergistic actuation of the brace was fulfilled with one type of actuator. By realizing local curvatures at each side of wrist, this approach allows the brace to better accommodate the consecutive bending of the human wrist. Thus, better wearing comfort was achieved.
3) The proposed soft robotic wrist brace design achieved compact form factor while achieving 2-DoF wrist motion. Although based on a commercially available wrist brace and mass-fabricated origami soft actuators through blow molding, the SR wrist brace achieved up to 2 degrees of bending, 7.5 N force, and keeping a compact form factor of 160 mm*110 mm*85 mm. The range of motion (*θ*_f_ = 31°, *θ*_e_ = 30°, *β*_r_ = 33°, *β*_u_ = 22°) meets the required functional range listed in Palmer et al., (1985), i.e., 5° of flexion, 30° of extension, 10° of radial deviation, and 15° of ulnar deviation. According to Flores et al. (2014), the average maximal torque generated by the wrist of a healthy individual in radial/ulnar deviation is 1.3 Nm. As shown in Figure 7F, the force generated from proposed SR brace in radial/ulnar deviation was around 6 N; thus, considering the length of the 2-actuator unit of 128 mm, the torque generated by the SR brace reached up to 0.76 Nm, which is about 58% of the max torque of human wrist.

Although the proposed SR brace is not developed for specific use case, the performance is comparable with existing devices of certain purpose. For example, the carpal tunnel syndrome soft relief device in Zhu et al. (2017) recorded a 2 cm lift of the wrist, which is higher than a measured keyboard height (1.9 cm), to provide more comfort and relief of the syndrome for the user. In comparison, the proposed SR brace is able to move the wrist to 30° in flexion; with its length of 16 cm, the brace can lift the wrist up to 8 cm, which means its range of motion is sufficient in this use case. According to the results shown in Figure 9D, the bandwidth of the SR is adequate to perform mirror therapy rehabilitation, where the human-in-the-loop response dynamics with visual tracking is around 0.5 Hz (Morris et al., 2017). However, the brace can only generate 58% of the max torque of human wrist in radial/ulnar deviation (Flores et al., 2014). This can be improved by increasing the thickness of the SOA facets to allow higher inflation pressure in its cavity.

### Conclusions and Future Work

In this work, a compact and low-profile soft robotic wrist brace was developed using a modular approach. Eight modular soft origami actuators were arranged in four identical units and attached to a commercially available wrist brace. The linear motion of the SOA was defined by the variated Yoshimura origami pattern with trapezoid facets. The SOAs were made of EVA by blow molding. The repeatability of the mass-produced SOAs was verified by experiments, which verified their practicality of fabricating soft actuators in large quantity. When installed on the wrist brace, the axial deformations of the actuators were constrained by the fabrics and derived bending deformation. To transmit the force of the actuators to the brace, rigid anchors were used to connect the ends of the actuators to the brace. Thus, the SR wrist brace generated the 2-DoF motions of the wrist joint, i.e., extension/flexion, ulnar/radial deviation, with soft and rigid components.

The actuation and control system of the soft robotic wrist brace was presented in this work, with the performance tested. The experiments on the mechanical performance, including the range of motion, output force, wearing position adaptivity, and performance under loading, were carried out with results analyzed. The synchronistic approach to actuate the brace was demonstrated in the experiments, showing the superior performance in generating output force and angular displacement. The findings in this work proved the soft robotic wrist brace satisfied the functional range of human wrist. Thus, the potential to be applied as the wrist rehabilitation device was displayed.

Future works include developing a compact wearable soft robotic brace system which integrated the control systems; further improving the output force and range of motion for broader application scenarios, such as human joint augmentation and motion capture; carrying out user case studies to validate the effectiveness and side effects of specific rehabilitation therapies; optimizing the design of the brace to reduce the volume and weight to achieve a nonobstructive wearing experience in daily living environment; applying the modular actuator approach to construct wearable devices for other joints of human limbs.

## Data Availability

The original contributions presented in the study are included in the article/Supplementary Material; further inquiries can be directed to the corresponding author.
